# Supplementary material for: COZOID: contact zone identifier for visual analysis of protein-protein interactions
Source: BMC Bioinformatics. 2018 Apr 6;19:125. doi: 10.1186/s12859-018-2113-6 (PMC5889581; doi:10.1186/s12859-018-2113-6)
Supplement: Supplementary file 2 — Software build. Executable binary file of the software tool. (ZIP 118,784 kb) [file 12859_2018_2113_MOESM2_ESM.zip › cozoid/java/docs/beansbinding-1.2.1-doc.zip!/org/jdesktop/el/ELContextEvent.html]

ELContextEvent (Beans Binding)


---


|  |  |  |
| --- | --- | --- |
| **Overview** \| **Package** \| **Class** \| **Use** \| **Tree** \| **Deprecated** \| **Index** \| **Help** | |  |
| **PREV CLASS**   **NEXT CLASS** | **FRAMES**    **NO FRAMES**     **All Classes** |  |
| SUMMARY: NESTED \| FIELD \| CONSTR \| METHOD | DETAIL: FIELD \| CONSTR \| METHOD |  |


---


## org.jdesktop.el Class ELContextEvent

```
java.lang.Object
  java.util.EventObject
      org.jdesktop.el.ELContextEvent
```

**All Implemented Interfaces:**: java.io.Serializable

---

``` public class ELContextEvent extends java.util.EventObject ```

An event which indicates that an `ELContext` has been created.
The source object is the ELContext that was created.

**Since:**
:   JSP 2.1

**See Also:**: `ELContext`, `ELContextListener`, Serialized Form

---

**Field Summary**

**Fields inherited from class java.util.EventObject**

`source`


**Constructor Summary**

`ELContextEvent(ELContext source)`
  
          Constructs an ELContextEvent object to indicate that an
`ELContext` has been created.


| **Method Summary** | |
| --- | --- |
| `ELContext` | `getELContext()`             Returns the `ELContext` that was created. |

**Methods inherited from class java.util.EventObject**

`getSource, toString`

**Methods inherited from class java.lang.Object**

`clone, equals, finalize, getClass, hashCode, notify, notifyAll, wait, wait, wait`

**Constructor Detail**

### ELContextEvent

```
public ELContextEvent(ELContext source)
```

:   Constructs an ELContextEvent object to indicate that an
    `ELContext` has been created.

    **Parameters:**: `source` - the `ELContext` that was created.


**Method Detail**

### getELContext

```
public ELContext getELContext()
```

:   Returns the `ELContext` that was created.
    This is a type-safe equivalent of the `EventObject.getSource()` method.

    :   **Returns:**: the ELContext that was created.


---


|  |  |  |
| --- | --- | --- |
| **Overview** \| **Package** \| **Class** \| **Use** \| **Tree** \| **Deprecated** \| **Index** \| **Help** | |  |
| **PREV CLASS**   **NEXT CLASS** | **FRAMES**    **NO FRAMES**     **All Classes** |  |
| SUMMARY: NESTED \| FIELD \| CONSTR \| METHOD | DETAIL: FIELD \| CONSTR \| METHOD |  |


---
